# Brain-Computer Interface for Control of Wheelchair Using Fuzzy Neural Networks

**DOI:** 10.1155/2016/9359868

**Published:** 2016-09-29

**Authors:** Rahib H. Abiyev, Nurullah Akkaya, Ersin Aytac, Irfan Günsel, Ahmet Çağman

**Affiliations:** ^1^Department of Computer Engineering, Applied Artificial Intelligence Research Centre, Near East University, Lefkosa, Northern Cyprus, Mersin 10, Turkey; ^2^Applied Artificial Intelligence Research Centre, Robotics Research Lab, Near East University, Lefkosa, Northern Cyprus, Mersin 10, Turkey

## Abstract

The design of brain-computer interface for the wheelchair for physically disabled people is presented. The design of the proposed system is based on receiving, processing, and classification of the electroencephalographic (EEG) signals and then performing the control of the wheelchair. The number of experimental measurements of brain activity has been done using human control commands of the wheelchair. Based on the mental activity of the user and the control commands of the wheelchair, the design of classification system based on fuzzy neural networks (FNN) is considered. The design of FNN based algorithm is used for brain-actuated control. The training data is used to design the system and then test data is applied to measure the performance of the control system. The control of the wheelchair is performed under real conditions using direction and speed control commands of the wheelchair. The approach used in the paper allows reducing the probability of misclassification and improving the control accuracy of the wheelchair.

## 1. Introduction

Human brain control of wheelchairs for physically disabled people has attracted great attention due to their convenience and relatively low cost, high mobility, and quick setup. The measurement of human brain signals and converting them into control signals require the development of an interface between the brain and the computer. A brain-computer interface (BCI) system provides communication between computer and mind of pupils. This interface can be based on brain activity during muscular movements or the changes of the rhythms of brain signals [1]. These brain activities can be detected using electroencephalographic (EEG) signals. BCI transforms the EEG signals produced by brain activity into control signals which can be later used for controlling the wheelchair without using any physical controls. Since the brain signals are very weak, we need to apply amplifiers and some spatial and spectral filters to the EEG signals in order to extract the features of these signals. The detected EEG signals are based on the change of frequencies and change of amplitudes. For example, during voluntary thoughts, the frequencies of signals change, and during movement, synchronisation/desynchronisation of brain activity which involves *μ* rhythm amplitude change happens. This relevant characteristic makes rhythm based BCI suitable to be used.

Recently, some research works have been done to develop many applications of BCI for wheelchairs. The main function of BCI is to convert and transmit human intentions into appropriate motion commands for the wheelchairs, robots, devices, and so forth. BCI allows improving the quality of life of disabled patients and letting them interact with their environment. Reference [2] presents the application of BCI and control of wheelchair in an experimental situation. The research considers the driving of a simulated wheelchair in a virtual environment before using BCI in a real situation. The virtual reality (VR) decreases the number of dangerous situations by using training and testing applications. Reference [3] describes a BCI system which controls the wheelchair that moves in only one direction: move forward. In [4, 5], BCI is designed for control of wheelchair using three possible commands: turn left, turn right, and move forward. In [6], BCI is designed using EEG signal captured by eight electrodes. Wavelet transform was used for feature extraction and the radial basis networks were used to classify the predefined movements. In [7], controller based on the brain-emotional-learning algorithm is used to control the omnidirectional robot. Reference [8] presents the design of an asynchronous BCI based control system for humanoid robot navigation using an EEG. Reference [9] considers a noninvasive EEG-based brain-computer interface system to achieve stable control of a low speed unmanned aerial vehicle for indoor target searching. References [10–15] consider the design of brain-controlled wheelchair. The construction of viable brain-actuated wheelchair that combines BCI with a commercial wheelchair, via a control layer, is considered. Combining the BCI with shared control architecture [11] allows for dynamically producing intuitive and smooth trajectories. The processes of feature extraction and classification are very important in BCI design and they have a great effect on the performance of the BCI system. Set of research works has been done for improvement of the feature extraction and classification algorithms [12–19]. References [16, 17] consider feature extraction algorithms for BCI. Reference [17] uses adaptive common spatial patterns for feature extraction.

Different clustering algorithms based on support vector machines, linear discriminant analysis, and neural networks are applied for classification of brain signals. Reference [18] uses features, optimised in the sense of statistically significant and potentially discriminative coherences at a specific frequency, and applies linear discriminant analysis (LDA) for classification purpose. SVM [19] and LDA [20] are used for classification purpose of brain signals.

Recently, several soft computing techniques are used for recognition of brain activity [21–23]. Reference [21] uses fuzzy logic and [22] uses neural networks with fuzzy particle swarm optimisation for BCI design. In [23], continuous wavelet transform is used to extract highly representative features and then an Adaptive Neuron-Fuzzy Inference System (ANFIS) is applied for classification. The systems based on fuzzy logic can make classifications using vague, imprecise, noisy, or missing input information. On given problem, human perception process can be efficiently modelled using fuzzy logic.

As shown, feature extraction and classification play an important role in the design of brain-based control for obtaining high classification accuracy. In the BCI design, high classification rate is very important. Otherwise, the presence of errors can cause initiation of a wrong command that can lead to dangerous situations. Therefore, achieving low error rates keeps the users safe. Different clustering algorithms based on support vector machines, linear discriminant analysis, and neural networks are applied for classification of brain signals [18]. Fuzzy classification represents knowledge more naturally to the way of human thinking and is more robust in tolerating imprecision, conflict, and missing information. In this paper, the fuzzy neural network structure is proposed for the design of BCI in order to achieve efficient brain-based control of wheelchair. In the literature, different neural and fuzzy networks are proposed for solving various problems [23–29]. In [23–26], FNN structure is designed for control of dynamic plants. In [27–29], neurofuzzy inference systems are designed for classification and control purposes. The systems designed in these papers are used for special purposes. In the neurofuzzy structures, the rules are constructed using all possible combinations of inputs and cluster centres. The problems that are characterised by multiple inputs and multiple outputs will have a huge number of fuzzy rules. The constructions of such systems are not efficient and these systems have a huge number of parameters. In this paper, the number of rules is selected using the clustering results which is equal to the number of cluster centres. In this paper, in order to improve the performance of classification system, a multi-input and multioutput fuzzy neural system (FNS) based on TSK rules is proposed for classification of the ECG signals.

The paper is organised as follows.  Section 2 presents the architecture of BCI system based on FNN. Signal processing and feature extraction stages have been described.  Section 3 presents classification algorithm based on FNN.  Section 4 presents parameter updates rule used for FNN. The fuzzy *c* means classification and gradient descent algorithms are applied for updating parameters of FNN.  Section 5 gives experimental results obtained for FNN based BCI system.  Section 6 presents conclusions of the paper.

## 2. BCI System Architecture

**Figure 1:**
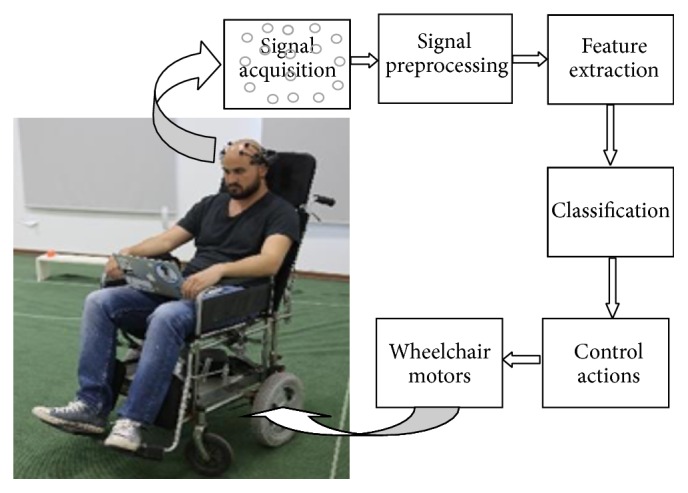
The BCI based control of the wheelchair.

Figure 1 depicts BCI based control of the wheelchair. BCI system consists of an Emotiv headset connected to a computer. Emotive sensors supply information to the computer. The computer runs the signal processing and classification algorithms and is connected to a microcontroller that controls the movement of the wheelchair. The wheelchair can move in four directions. The speed of the wheelchair is taken as constant and the wheelchair can be switched on and off in the case of necessity. Taking into account the abovementioned functionality, the BCI system uses the following commands: move forward, move backward, turn left, turn right, and turn on and turn off the switch.

**Figure 2:**
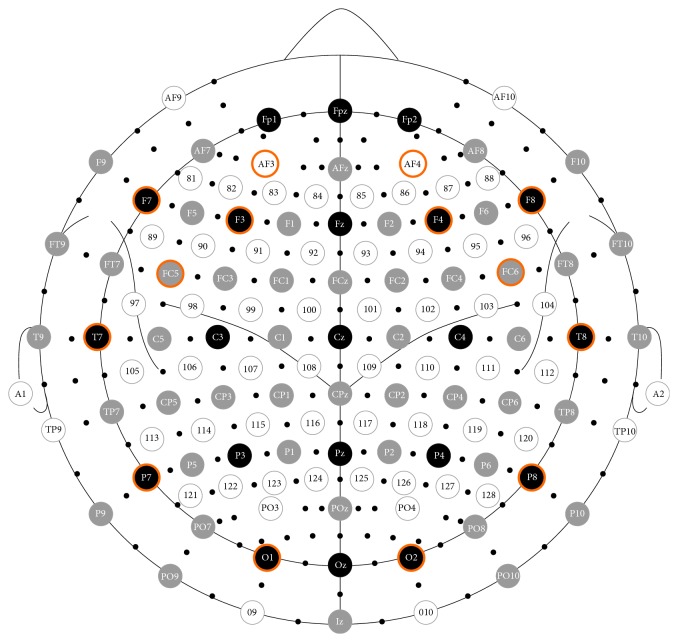
Emotiv's sensor layout compared to standard 72 sensors' layout. The distribution of EEG electrodes. Fourteen channels are marked for data acquisition.

A BCI based control system is usually composed of five main units: signal acquisition unit, signal preprocessing unit, feature extraction unit, classification unit, and action unit that controls motors of the wheelchair. The main units of the decision system are represented in Figure 1. In signal acquisition block, the EEG signals are captured using the Emotiv headset. Emotiv EPOC is an EEG headset which supplies 14-channel EEG data (Figure 2) and 2 gyros for 2-dimensional controls. Its features are adequate for a useful BCI (resolution and bandwidth). Our system uses upper face gestures for actuation commands; since most Emotiv sensors are located in the frontal cortex, they are the most reliable signals to detect. The EEG input signals are sent to the signal preprocessing unit for filtering and scaling and sent to the feature extraction block. In this block, the basic features are extracted and sent to the classification system. The classification block processes the input signals and outputs the control instructions. Later, these control instructions are sent to the motors of the wheelchair.

**Figure 3:**
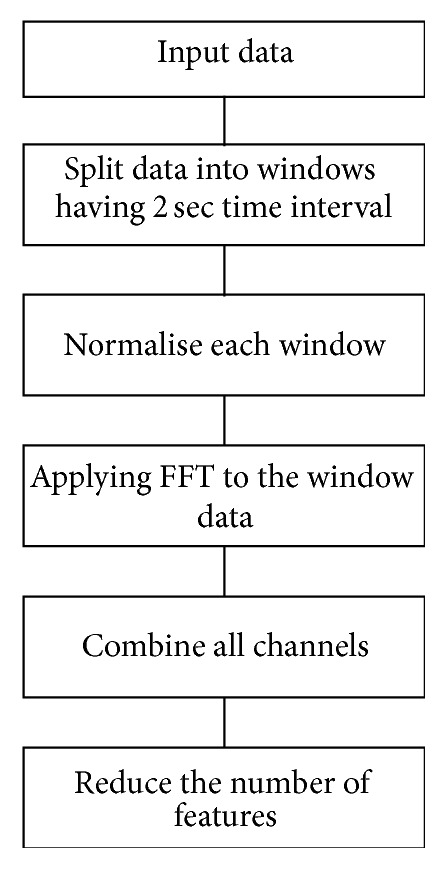
Signal preprocessing and feature extraction.

The EEG signals measured by Emotiv headset are first processed by signal preprocessing and feature extraction blocks. Signal preprocessing block filters the noises and scales the signals in a certain interval. These signals are very long and need certain time for processing. Therefore, the feature extraction technique is applied in order to decrease the signal size and extract more important features for classification. In the paper, we used fast Fourier transform (FFT) for extraction of the features of the input EEG signals.  Figure 3 presents the operations used in the feature extraction stage. The input signal received from the headset is divided into windows having 2 sec time interval with 50% overlap. The use of overlapping windows allows us to increase the accuracy of the classification. Each two-second window corresponds to 256 samples of data. Each second headset returns 128 data samples. The obtained signals from the channels, stored as windows, are then sent to normalisation block. Each channel is normalised in order to centre each channel on zero by calculating the mean value of each channel for the window and then subtracting it from each of the data points in the channel. After normalisation, Hamming window is applied to each channel in the window. EEG signals do not generally repeat exactly, over any given time interval, but the math of the Fourier transform assumes that the signal is periodic over the time interval. This mismatch leads to errors in the transform called spectral leakage. Hamming window is used to mitigate this problem. Then, fast Fourier transform (FFT) is applied to each channel in the window to find out the frequency components of the signal. Each frequency component is used as a feature, which results in 64 × 14 features. In order to increase the performance of the classification, the features are ranked by evaluating the worth of a frequency by measuring the information gain with respect to the class. The expected information gain is the change in information entropy (*H*) from a prior state to a state that takes some information as given.

Information gain selects a subset of the original representation attributes according to Information Theory quality metric. This method computes the value of the metric for each attribute and ranks the attributes. Then, it simply decides a threshold in the metric and keeps the attributes with a value over it.

After frequency representation, all channels in the window are combined into a single unit so as to apply classification on all channels at once. The filtering operation is applied in order to select important features of the brain signals. These features are used for classification purpose.

Besides the above-described approach, we can use also another approach for signal processing. In the second approach, the acquired brain signal after windowing, normalisation, and combining operations is used for classification purpose:

(1) InfoGainClass,Frequency=HClass−HClass ∣ Frequency.

In the paper, we use frequency representation of signals for classification. These signals are processed and classified. The output of classification system is used to control the wheelchair. Even though during training system reports 100% success rate in real-world conditions, it does misclassify, a state machine is used to further increase safety and reduce misclassification. As an example, the system will not transition from forward motion to backward motion without stopping in neutral. The output of the state machine drives the microcontroller which controls the motors on the wheelchair. The number of classes is equal to the number of control actions.

## 3. FNN Based Classification 

The features extracted from the EEG signals are used for classification and determining control action. In this paper, we propose a novel approach which is based on FNN for the classification of brain signals. The extracted features are input signals of the FNN based classifier. The classifier based on the extracted features classifies the signals into the following six classes: move forward, move backward, turn left, turn right, and turn on and turn off the switch. The design of FNN includes the development of the fuzzy rules that have IF-THEN form. This is implemented by dint of optimal definition of the premise and consequent parts of fuzzy IF-THEN rules for the classification system through training of fuzzy neural networks. In the paper, the Takagi-Sugeno-Kang (TSK) types of IF-THEN rules that have a fuzzy antecedent and crisp consequent parts are used. The TSK-type fuzzy system approximates nonlinear system with linear systems and has the following form:

(2) If x1  is  A1j  and  x2  is  A2j  and  …  and  xm  is  AmjThen yj  is  ∑i=1maijxi+bj.

Here, *x*_*i*_ and *y*_*j*_ are input and output signals of the system, respectively, *i* = 1,…, *m* is the number of input signals, and *j* = 1,…, *r* is the number of rules. *A*_*ij*_ are input fuzzy sets; *b*_*j*_ and *a*_*ij*_ are coefficients.

**Figure 4:**
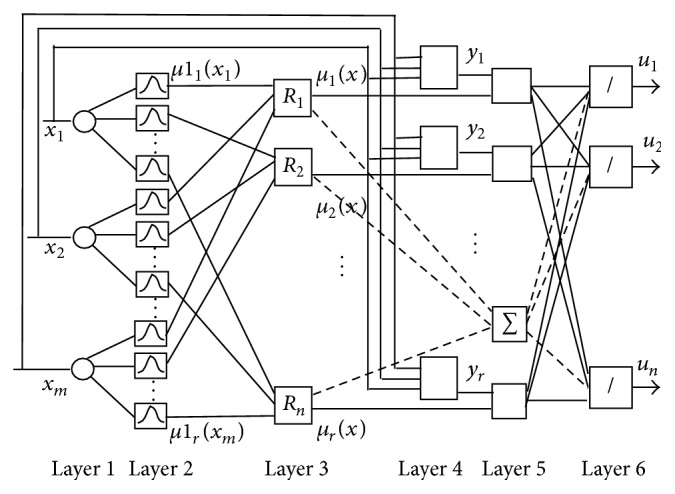
FNN based identifier.

The structure of fuzzy neural networks used for the classification of EEG signal is based on TSK-type fuzzy rules and is given in Figure 4. The FNN consists of six layers. The first layer is used to distribute the *x*_*i*_  (*i* = 1,…, *m*) input signals. The second layer includes membership functions. Here, each node represents one linguistic term. Here, for each input signal entering the system, the membership degree where input value belongs to a fuzzy set is calculated. In the paper, the Gaussian membership function is used to describe linguistic terms.

(3) $$\begin{array}{c} \mu 1_{j}^{}\left(\;x_{i}\;\right)=e^{-{\left(x_{i}-c_{ij}\right)}^{2}/\sigma_{ij}^{2}},\;i=1,\ldots ,m,\;\;j=1,\ldots ,r, \end{array}$$

where *c*_*ij*_  and  *σ*_*ij*_ are centre and width of the Gaussian membership functions, respectively. *μ*1_*j*_(*x*_*i*_) is membership function of the *i*th input variable for the* j*th term.* m* is a number of input signals;* r* is a number of fuzzy rules (hidden neurons in the third layer).

The third layer is a rule layer. Here, the number of nodes is equal to the number of rules. Here, *R*_1_, *R*_2_,…, *R*_*r*_ represents the rules. The output signals of this layer are calculated using t-norm min (AND) operation:

(4) $$\begin{array}{c} \mu_{j}^{}\left(\;x\;\right)=\prod_{i}\mu 1_{j}\left(\;x_{i}\;\right),\;i=1,\ldots ,m,\;\;j=1,\ldots ,r, \end{array}$$

where ∏ is the min operation.

These *μ*_*j*_(*x*) signals are input signals for the fifth layer. The fourth layer is a consequent layer. It includes *n* linear systems. Here, the values of rules output are determined as

(5) $$\begin{array}{c} y_{j}=\overset{m}{\underset{i=1}{\sum }}x_{i}w_{ij}+b_{j}. \end{array}$$

In the next fifth layer, the output signals of the third layer are multiplied by the output signals of the fourth layer. The output of *j*th node is calculated as

(6) $$\begin{array}{c} y1_{j}=\mu_{j}\left(\;x\;\right)\cdot y_{j}. \end{array}$$

In the sixth layer, the output signals of FNN are determined as

(7) $$\begin{array}{c} u_{k}=\frac{\sum_{j=1}^{r}w_{jk}y1_{j}}{\sum_{j=1}^{r}\mu_{j}\left(x\right)}. \end{array}$$

Here, *u*_*k*_ are the output signals of FNN (*k* = 1,…, *n*). After calculating the output signal, the training of the network starts.

The design of FNN (Figure 4) includes determination of the unknown parameters that are the parameters of the antecedents *c*_*ij*_ and *σ*_*ij*_ (*i* = 1,…, *m*, *j* = 1,…, *r*) and the consequents *w*_*jk*_, *a*_*ij*_, *b*_*j*_ (*i* = 1,…, *m*, *j* = 1,…, *r*, *k* = 1,…, *n*) parts of the fuzzy IF-THEN rules (2). In the next section, the training of the parameters of FNN is presented.

## 4. Parameter Updates

In the fuzzy IF-THEN rules (2), the antecedent part represents the input space by dividing the space into a set of fuzzy regions and the consequent part describes the system behaviour in those regions. In the design of FNN model, the basic problem is the determination of the unknown parameters of antecedent and consequent parts. Recently, a set of different approaches has been applied for designing fuzzy IF-THEN rules. These are clustering [30–35], gradient algorithms [24–27, 34–36], the least-squares method (LSM) [27, 33], and genetic algorithms [27, 34].

In this paper, the fuzzy clustering and gradient descent algorithms are applied for determining the parameters of FNN. The basic parameters of the antecedent part are the centres and widths of the membership functions. Learning of FNN starts with the update of parameters of antecedent part of IF-THEN rules, that is, the parameters of the second layer of FNN (Figure 4). For this purpose, FCM is applied in order to partition input space and construct antecedent part of fuzzy IF-THEN rules. In the result of partitioning the cluster centres are determined. These centres correspond to the centres of the membership functions used in the input layer of FNN. Using the distances between the cluster centres, the widths of the membership functions are determined.

After finding the parameters of the antecedent's parts, the design of the consequent part of the fuzzy rules starts. For this purpose, the gradient descent algorithm is applied for the parameter update of the consequent part, that is, the parameters of the fourth layer of FNN. In learning of FNN, 10-fold cross validation is applied for separation of the data into training and testing set.

The initial values of the parameters of consequent parts are generated randomly. The training of the parameters has been carried out using errors calculated on the output of the network. For generality, we have given the learning procedure of all parameters of FNN using gradient descent algorithm with adaptive learning rate. The adaptive learning rate used guarantees the convergence and speeds up the learning process. In addition, the momentum is also used to speed up the learning processes.

The error in the output of the network is calculated as

(8) $$\begin{array}{c} E=\frac{1}{2}\overset{n}{\underset{k=1}{\sum }}{\left(\;u_{k}^{d}-u_{k}\;\right)}^{2}. \end{array}$$

Here, *n* is the number of output signals of the network; *u*_*k*_^*d*^  and  *u*_*k*_ are desired and current output values of the network (*k* = 1,…, *n*), respectively. The parameters *w*_*jk*_, *a*_*ij*_, *b*_*j*_ (*i* = 1,…, *m*, *j* = 1,…, *r*, *k* = 1,…, *n*) in consequent part of network and the parameters of membership functions *c*_*ij*_ and *σ*_*ij*_ (*i* = 1,…, *m*, *j* = 1,…, *r*) in the premise part of FNN structure are adjusted as

(9) wjkt+1=wjkt−γ∂E∂wjk+λwjkt−wjkt−1;aijt+1=aijt−γ∂E∂aij+λaijt−aijt−1;bjt+1=bjt−γ∂E∂bj+λbjt−bjt−1;

(10) cijt+1=cijt−γ∂E∂cij+λcijt−cijt−1;σijt+1=σijt−γ∂E∂σij+λσijt−σijt−1;i=1,…,m;  j=1,…,r;  k=1,…,n,

where* m* is the number of input signals of the network (input neurons) and *r* is the number of fuzzy rules (hidden neurons). *γ* is the learning rate; *λ* is the momentum.

The derivatives in (9) are computed as

(11) ∂E∂wjk∂E∂uk∂uk∂wjk=ukt−ukdt·y1j∑j=1nμj,∂E∂aij∂E∂uk∂uk∂y1j∂y1j∂yj∂yj∂aij=∑kukt−ukdt·wkjμjxi∑j=1nμj,∂E∂bj∂E∂uk∂uk∂y1j∂y1j∂yj∂yj∂bj=∑kukt−ukdt·wkjμj∑j=1nμj,here  i=1,…,m,  j=1,…,r,  k=1,…,n.

In (10), the derivatives are determined as

(12) ∂E∂cij=∑k∂E∂uk∂uk∂μj∂μj∂cij,∂E∂σij=∑k∂E∂uk∂uk∂μj∂μj∂σij.

Here, *i* = 1,…, *m*, *j* = 1,…, *r*, *k* = 1,…, *n*.

(13) ∂E∂uk=ukt−ukdt;∂uk∂μj=yj−uk∑j=1nμj;∂μjxi∂cij=μjxi2xi−cijσij2;∂μjxi∂σij=μjxi2xi−cij2σij3.

Using (11)–(13), the derivatives in (9) and (10) are calculated and the correction of the parameters of FNN is carried out.

## 5. Experiments and Results

**Figure 5:**
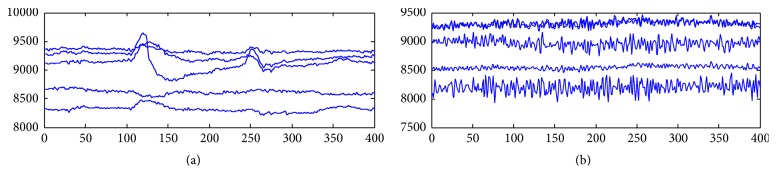
EEG signals for five channels: (a) neutral pose and (b) positive gesture pose.

The BCI system is simulated and used in real life applications. The EEG signals are measured with signal acquisition unit, the Emotiv EPOC headset. In the experiments, we have utilised 14 channels for measuring EEG signals. The measured EEG signals have different rhythms within the frequency band. The experiments show that measuring brain signals is difficult so we have tested our system using brain muscle signals. As an example, the signals obtained from 5 sample channels are shown in Figure 5.  Figure 5(a) depicts a neutral pose, patient relaxing and not doing anything.  Figure 5(b) depicts a positive gesture. As shown in the figures, the EEG signals with positive gesture pose are changing more frequently than a neutral pose. In the paper, the FFT is applied to extract important features of the signal. After the preprocessing stage, given in Section 2, the important features of these signals are extracted and used for classification purpose. The number of extracted features was determined as 100. These signals are inputs for FNN system. Outputs of FNN model are clusters. The following clusters are used in the experiment: move forward, move backward, turn left, turn right, and turn on and turn off the switch. For each cluster, the system recorded 10 seconds of data.

In this paper, the classification of the EEG signals is performed using FNN model. For this purpose, the FNN structure with hundred input and six output neurons is generated. In the papers [27, 29], the neurofuzzy systems have been efficiently applied for different classification problems. If we use these structures for 100 inputs and 2 cluster centres, 2^100^ rules should be generated. The rules are constructed using all possible combinations of inputs and cluster centres. This is a very large number. In this paper, the number of rules is selected according to the clustering results, equal to cluster centres.

Fuzzy *c* means classification is used in order to design the premise parts of (2) and to determine the parameters of Gaussian membership functions used in the second layer of FNN. In experiments, different cluster numbers, 5, 6, 9, and 16, are used to design FNN structure. These experiments have been done in order to increase the performance of classification system. At first, FCM clustering is used for the input space with 6 clusters for each input. Six fuzzy rules are designed using a different combination of these clusters for 100 inputs. After clustering input space gradient descent algorithm is applied for learning of consequent parts of the fuzzy rules, that is, parameters of the 4th layer of FNN. In learning of FNN, 10-fold cross validation is used for separation of the data into training and testing set.

**Figure 6:**
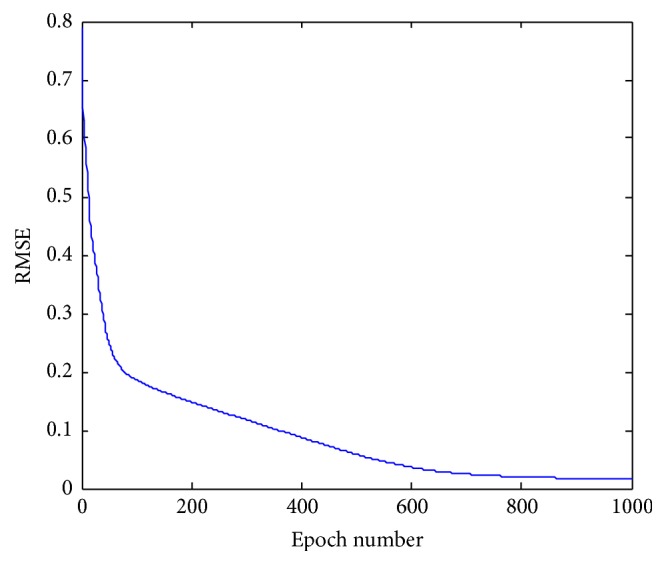
Training of FNN.

The initial values of the parameters of FNN are randomly generated in the interval [−1, 1] and, using the gradient algorithm derived above, they are updated for the given input-output training pairs. As a performance criterion, RMSE is used.  Figure 6 depicts the evolution of the RMSE values over 1000 epochs.

For training of the FNN, 1000 epochs are used. As a result of training, the values of the parameters of the FNN system were determined. Once the FNN has been successfully trained, it is then used for the classification of the EEG signals. During learning, the value of RMSE was obtained as 0.223264 for training data and 0.241625 for evaluation. After learning, for the test data, the value of RMSE was obtained as 0.257986 with 100% accuracy of classification.  Figure 6 depicts RMSE values obtained during training. The design of FNN model is performed using a different number of rules.  Table 1 includes results of simulations with 5, 6, 9, and 16 rules, respectively. As shown, accuracy of FNN classification model is 100%.

For comparison purpose, we test the system using different classification techniques. As a result of the classification, the following results are obtained (Table 2). In the table, the classification results of FNN model are compared against linear logistic regression model [36], SVM with various kernels, multilayer perceptron (MLP) with various hidden layers, Naïve Bayes classifier [37], Random Tree, and Random Forest [38]. As shown, the simulation results demonstrate the efficiency of application of FNN model in the classification of EEG signals. These clusters activate the corresponding control signal which is then used to actuate the motors of the wheelchair.

## 6. Conclusion

The paper presents the design of BCI based on FNN for a wheelchair. The emotional and muscular states of the user are evaluated for control purposes. The design of BCI has been done to actuate a brain-controlled wheelchair using six mental activities of the user: move backward, move forward, turn left, turn right, turn on, start, and stop. For classification of EEG signals, the FNN with 10-fold cross validation data set is used. The design of the FNN system is implemented using fuzzy *c* means classification and gradient descent algorithm. The obtained 100% classification results prove that the used techniques are a potential candidate for the classification of the EEG signals in the design of brain-based control system. In the future, we are going to improve the number of commands for control of wheelchair and decrease detection time of the EEG signal used for measuring brain activities and design efficient brain-controlled wheelchair.

## Figures and Tables

**Table 1:** Classification results.

Number of rules	Correctly classified instances	Incorrectly classified instances	Training RMSE	Evaluation RMSE	Test RMSE
5	92%	3	0.465492	0.464918	0.476516
6	100%	0	0.223264	0.241625	0.257986
9	100%	0	0.152714	0.153688	0.153874
16	100%	0	0.047268	0.048324	0.048262

**Table 2:** Comparison of classification results.

Method	Correctly classified instances	Incorrectly classified instances	Mean absolute error	Root mean squared error
Linear logistic regression model	96%	4%	0.0214	0.1265
SVM (polynomial kernel)	100%	0	0.24	0.3162
SVM (RBF kernel)	74%	26%	0.2568	0.3404
SVM (PUK kernel)	96%	4%	0.2424	0.32
MLP (NN) (5 hidden neurons)	88%	12%	0.0724	0.1586
MLP (NN) (6 hidden neurons)	100%	0	0.048	0.0958
Naïve Bayesian	94%	6%	0.024	0.1549
Random Tree	74%	26%	0.104	0.3225
Random Forest	98%	2%	0.1215	0.179
FNN (6 hidden neurons)	100%	0	1.823	0.257986
